# Transperineal Ultrasound Measurement of Retrovesical Angle at Rest for Assessing Severity of Stress Urinary Incontinence: A Cross-Sectional Study

**DOI:** 10.3390/jcm15145559

**Published:** 2026-07-15

**Authors:** José Pablo Traña-Serrano, Cristina Orts-Ruiz, Francisco Javier Molina-Payá, Sergio Montero-Navarro, José Martín Botella-Rico, Andrés Zamora-Streber, Jesús Sánchez-Más, Cristina Salar-Andreu

**Affiliations:** 1 Clínica Traña, Nunciatura 68A, Mata Redonda, San José 10203, Costa Rica; josetrana@clinicatrana.com (J.P.T.-S.); azamora2610@gmail.com (A.Z.-S.); 2 Departamento de Fisioterapia, Facultad de Ciencias de la Salud, Universidad Cardenal Herrera-CEU, CEU Universities, 03204 Elche, Alicante, Spain; cristina.orts@uchceu.es (C.O.-R.); francisco.molina@uchceu.es (F.J.M.-P.); sergio.montero@uchceu.es (S.M.-N.); jmbotella@uchceu.es (J.M.B.-R.); cristina.salar@uchceu.es (C.S.-A.); 3 Departamento de Ciencias Biomédicas, Facultad de Ciencias de la Salud, Universidad Cardenal Herrera-CEU, CEU Universities, 03204 Elche, Alicante, Spain

**Keywords:** stress urinary incontinence, severity, diagnosis, transperineal ultrasound, quality of life

## Abstract

**Background/Objectives**: The aim of this diagnostic accuracy study was to evaluate the usefulness of transperineal ultrasound parameters of urethral instability for assessing the severity of stress urinary incontinence (SUI) in women and their relationship with health-related quality of life (HRQOL). **Methods**: This cross-sectional study included 240 women with symptomatic SUI (mean age 41 years). Severity was classified using the Sandvik Severity Index (reference standard). Transperineal ultrasound (index test) was performed to measure the retrovesical angle (RVA) at rest and during Valsalva manoeuvre, bladder neck descent (BND), and urethral rotation angle (URA). Impact on HRQOL was assessed with the ICIQ-SF and King’s Health Questionnaire (KHQ). Operators were blinded to clinical data and vice versa. **Results**: The RVA at rest showed the best discriminatory capacity for identifying severe/very severe SUI (AUC 0.83, 95% CI 0.77–0.89; sensitivity 71%, specificity 84%), with an optimal cut-off value of 118.1° (adjusted for age, BMI and miscarriage). RVA values increased progressively with SUI severity both at rest and during Valsalva (*p* < 0.001). Significant positive correlations were found between RVA and ICIQ-SF scores (ρ^2^ = 0.412 at rest, *p* < 0.001). In contrast, URA remained within normal limits (<20°) and did not differ across severity groups. **Conclusions**: Transperineal ultrasound measurement of the RVA at rest is a reliable, non-invasive parameter for grading SUI severity and correlates with its impact on HRQOL. These findings suggest that RVA at rest has good discriminatory capacity for identifying women with severe or very severe SUI and may have potential as an adjunct to clinical assessment. However, given the moderate sensitivity observed and the lack of data on management outcomes, prospective studies are needed to determine its utility in guiding referral or treatment decisions.

## 1. Introduction

Stress urinary incontinence (SUI) is the most prevalent form of urinary incontinence in women and represents a significant public health concern [1,2,3]. It is defined as the involuntary leakage of urine on effort or physical exertion, or on sneezing or coughing, and arises primarily from two non-exclusive pathophysiological mechanisms: urethral hypermobility, in which the supportive structures fail to maintain the urethra in its correct anatomical position, and intrinsic sphincter deficiency, characterized by inadequate urethral coaptation and reduced urethral closure pressure [4,5,6].

Although SUI is not life-threatening, it exerts a profound negative impact on health-related quality of life (HRQOL), affecting daily activities, social participation, psychological well-being, and work productivity, and is strongly associated with anxiety, depression, and reduced self-esteem [3,7,8]. Greater severity of SUI has been consistently linked to a more pronounced deterioration in HRQOL [3,7,8].

The clinical diagnosis of SUI relies on medical history, physical examination, validated questionnaires, and, when indicated, urodynamic studies or imaging [9]. While these approaches are useful, they have notable limitations, including subjectivity in symptom reporting and the invasive nature of urodynamic testing.

Transperineal ultrasound has emerged as a non-invasive, real-time, and cost-effective imaging modality (Level of Evidence 2, Grade of Recommendation B) that allows detailed visualization of the bladder neck, urethra, and surrounding pelvic floor structures [4,10]. It enables quantitative assessment of urethral mobility through parameters such as bladder neck descent (BND), retrovesical angle (RVA, also known as the β-angle), and urethral rotation angle (URA) [11,12,13,14,15].

The existing literature has established that women with SUI generally exhibit greater BND and larger changes in RVA and URA compared with continent women [4,13,14,16]. However, most studies have focused on the binary diagnosis of SUI (presence versus absence) rather than on its graded severity. Consequently, the relationship between specific transperineal ultrasound parameters, particularly the bladder neck opening (RVA) measured at rest and during maximal Valsalva manoeuvre, and the clinical severity of SUI remains insufficiently characterized. Furthermore, data linking these ultrasound findings directly to the impact on HRQOL are limited. These gaps hinder the ability of clinicians to use transperineal ultrasound not only for diagnosis but also for objective severity stratification, which is essential for individualized treatment planning (conservative management versus surgical intervention).

The present study was therefore designed to evaluate the discriminatory capacity of transperineal ultrasound parameters of urethral instability, specifically the RVA at rest and during Valsalva, BND, and URA, in discriminating different severity grades of SUI, as classified by the Sandvik Severity Index (reference standard). A secondary aim was to examine the association between these ultrasound measures and the impact of SUI on HRQOL, as assessed by the ICIQ-SF and KHQ.

## 2. Materials and Methods

### 2.1. Study Design, Setting, Participants, and Sample Size

This cross-sectional observational study was designed as a single-center, cross-sectional observational investigation. Participants were consecutively recruited among women over 18 years of age who attended pelvic floor physiotherapy consultations for symptoms of SUI at the Traña Clinic (San José, Costa Rica) between 13 February and 14 May 2025.

During the initial consultation, potential participants received detailed information about the study, provided written informed consent, and underwent clinical screening to exclude visible scars, congenital malformations, abnormal pelvic masses, tissue irritation, or any other conditions that could affect pelvic floor or urinary function [17,18].

The women had to be over 18 years of age and meet the International Continence Society (ICS) diagnostic criteria for SUI, which consists of involuntary leakage of urine during increased intra-abdominal pressure in the absence of bladder contractions such as coughing, sneezing, and other physical activities. Exclusion criteria comprised the following: diagnosis of urge urinary incontinence or mixed urinary incontinence, pelvic organ prolapse (any stage), current or previous treatment for urinary incontinence, pregnancy, history of pelvic surgery, active urinary tract infection, or diagnosed psychological disorders.

Sample size was estimated on the basis of previous studies evaluating urethral mobility in SUI, which reported moderate effect sizes for ultrasound parameters such as the RVA. Using data from Dong et al. (mean difference of 9.9° during Valsalva manoeuvre; Cohen’s d ≈ 0.62), we calculated that approximately 48 participants per severity group would be required to detect a minimum effect size of d = 0.5 with 80% power and a two-tailed α= 0.05. Considering four severity levels and an expected distribution of approximately 85% mild/moderate cases, the target sample was set to ensure adequate representation across groups.

### 2.2. Data Collection and Procedure

Data collection was structured to ensure temporal separation between the index test (transperineal ultrasound) and the reference standard (Sandvik Severity Index), with both performed during the second consultation. Participants first completed a self-administered Google Form in the presence of a trained evaluator who was available to clarify questions without influencing responses. The form collected anthropometric data (age, body mass index), lifestyle habits (smoking, alcohol, and recreational drug use), and obstetric-gynaecological history (number of full-term pregnancies, mode of delivery, episiotomy, perineal laceration, and miscarriage). Menopausal status, current or past use of hormonal therapy (systemic or local), detailed lifetime history of pelvic floor muscle training (beyond the exclusion of recent treatment), and the presence of chronic respiratory disease or chronic cough were not systematically recorded.

Severity of SUI was assessed using the Sandvik Severity Index (reference standard), a validated two-item questionnaire that classifies incontinence as mild (score 1–2), moderate (3–6), severe (8–9), or very severe (12). This instrument demonstrates good criterion validity against pad-weighing tests and high test–retest reliability [19]. Assessors of the reference standard had no access to clinical details or ultrasound findings. The Sandvik Severity Index was selected as the reference standard because it is a simple, validated, and widely accepted tool specifically developed for epidemiological and clinical assessment of urinary incontinence severity in women. It has shown good criterion validity when compared with objective pad-weighing tests [19] and provides a clinically relevant, patient-reported classification of severity that correlates with impact on daily life and HRQOL. This facilitates translation of ultrasound findings into practical management decisions in a pelvic floor physiotherapy setting. While urodynamic testing remains the gold standard for confirming the diagnosis and mechanism of SUI in selected cases, and pad testing offers objective quantification of leakage, these methods are more invasive or burdensome; therefore, a validated symptom severity index was considered the most appropriate pragmatic reference standard for this study. This pragmatic choice aligns with the study setting (pelvic floor physiotherapy clinic) and the aim of evaluating a non-invasive ultrasound tool for severity stratification in routine practice.

Impact on HRQOL was evaluated with two validated self-reported instruments:

- Short International Consultation on Incontinence Quality Questionnaire (ICIQ-SF): This is a self-administered questionnaire with four questions and a total score ranging from 0 [best] to 21 [worst], which identifies people with urinary incontinence and its impact on their HRQOL [20].
- King’s Health Questionnaire (KHQ): KHQ is a self-administered assessment tool specifically designed to assess the HRQOL of women with Urinary incontinence (UI). It consists of 21 items distributed across nine dimensions: perception of overall health status (1 item), impact of UI (1 item), limitations in daily activities (2 items), physical limitations (2 items), social limitations (2 items), personal relationships (3 items), emotions (3 items), sleep/energy (2 items) and symptom severity scale (5 items). The score for each dimension ranges from 0 (lowest impact of UI and therefore best HRQOL) to 100 (highest impact, worst HRQOL). This questionnaire provides a specific value for each dimension on a scale with the following range: 0—Best possible HRQOL; 100—Worst possible HRQOL [21].

### 2.3. Ultrasound Scan (Index Test)

Transperineal ultrasound was performed using a MyLab X8 ultrasound system (Esaote, Florence, Italy) equipped with a 3.5–6 MHz convex transducer. Participants were instructed to attend with a comfortably full bladder (approximately 150–250 mL) to optimize visualization of the bladder neck and vaginal wall support. Participants were instructed to completely empty their bladder immediately before the ultrasound examination, in accordance with the standardized translabial/transperineal ultrasound protocol described by Dietz [22]. Following voiding, post-void residual volume was objectively assessed by ultrasound, and detrusor wall thickness was measured as part of the standard functional pelvic floor evaluation. This approach was adopted to minimize the potential confounding influence of bladder volume on measurements of urethral mobility, bladder neck descent, urethral rotation, and retrovesical angle. All examinations were conducted with the patient in the dorsal lithotomy position (hips flexed and slightly abducted, heels close to the buttocks), which is the standard and most recommended position for this type of assessment [22].

The transducer, covered with a sterile probe cover and acoustic gel, was placed longitudinally on the perineum in the midsagittal plane to obtain a clear view of the pubic symphysis, urethra, bladder neck, and posterior bladder wall [23]. All images were acquired at rest and during maximum Valsalva manoeuvre (sustained for at least 5–6 s). In all examinations, the best Valsalva attempt was chosen as the image showing the greatest caudal displacement of the bladder neck, while strictly maintaining a stable probe position and a sustained, effective strain. Specifically, selection was based on (1) clear visualization of the pubic symphysis, urethra and bladder neck; (2) absence of visible transducer movement or compression artefacts; and (3) the largest bladder neck excursion achieved over the 5–6-s Valsalva among the at least three attempts, as judged by the operator following a standardized protocol. The researchers performing and analyzing the ultrasound examinations were blinded to clinical data and reference standard results.

The following standardized parameters were measured offline on frozen midsagittal images, using the inferoposterior margin of the pubic symphysis as the main anatomical reference [24,25,26,27]:

- Bladder–symphysis distance (BSD): The perpendicular distance (in mm) from the inferoposterior margin of the pubic symphysis to the bladder neck (urethrovesical junction).
- BND: The difference in BSD between maximum Valsalva and rest (BND = BSD at Valsalva—BSD at rest), expressed in mm. Values >20 mm were considered indicative of significant hypermobility.
- RVA (also known as β-angle): The angle (in degrees) formed between the proximal urethral axis and a line drawn tangent to the posterior bladder base near the bladder neck. RVA was measured both at rest (RVA at rest) and during maximum Valsalva (RVA at Valsalva).
- ΔRVA: The change in RVA (ΔRVA = RVA at Valsalva—RVA at rest), representing dynamic opening of the bladder neck.
- URA: The change in the inclination angle of the proximal urethra relative to the vertical (or central symphyseal) axis between rest and maximum Valsalva (URA = α at Valsalva—α at rest), expressed in degrees. Values < 20° were considered within the normal range in this study.

**Figure 1:**
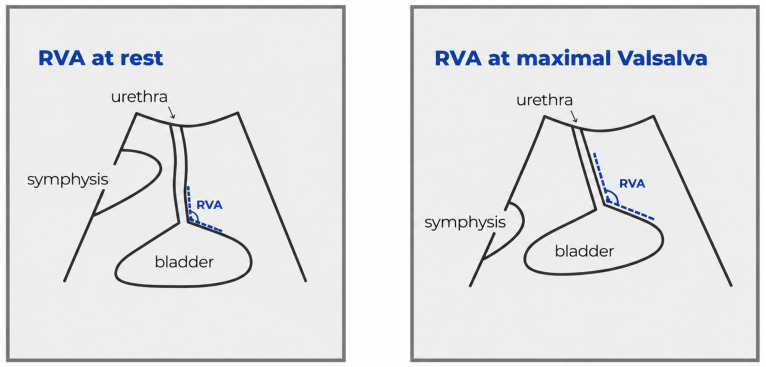
Schematic illustration of retrovesical angle (RVA, β-angle) measurement by transperineal ultrasound in the mid-sagittal plane. (**Left**) At rest. (**Right**) During Valsalva manoeuvre. Note the increase in RVA and bladder neck opening during straining.

Figure 1 shows a schematic illustration of the RVA measurement by transperineal ultrasound in the mid-sagittal plane, both at rest and during the Valsalva manoeuvre. The RVA is formed by the proximal urethral axis and a tangent to the posterior bladder base near the bladder neck. During Valsalva, an increase in this angle reflects bladder neck opening and urethral hypermobility.

All ultrasound measurements were performed offline on frozen midsagittal images by a single experienced operator. Previous validation studies have demonstrated excellent intra- and inter-observer reliability for the transperineal ultrasound parameters used in this study (BND, RVA, and URA) in women with SUI. Reported intraclass correlation coefficients range from 0.86 (95% CI 0.78–0.96) for BND, 0.91 (95% CI 0.83–0.97) for the RVA, and 0.92 (95% CI 0.85–0.97) for the URA [26,27]. Intra-observer reliability was not formally reassessed in the current cohort, as all measurements were performed by a single highly experienced operator (>5 years in pelvic floor ultrasound), and previous validation studies have consistently demonstrated excellent reproducibility for these parameters [26,27]. Inter-observer reliability was not evaluated because a single operator performed all analyses. The researcher responsible for performing the ultrasound did not have access to clinical information and standard reference results.

### 2.4. Statistical Analysis

Data are presented as mean ± standard deviation for continuous variables and as number (percentage) for categorical variables. Normality of distribution was assessed using the Kolmogorov–Smirnov test. Given the non-normal distribution of most variables, between-group comparisons across the four severity categories (defined by the Sandvik Severity Index) were performed using the non-parametric Kruskal–Wallis test for continuous variables and Pearson’s χ^2^ test for categorical variables. Post hoc power analyses were performed using GPower (version 3.1.9.7) [28]. A sensitivity analysis merging the severe and very severe categories was also conducted to assess the robustness of the between-group comparisons.

Spearman’s rank correlation coefficient (ρ) was used to evaluate the relationship between ultrasound parameters, SUI severity, and quality-of-life scores.

To assess the diagnostic performance of the ultrasound parameters for discriminating severe/very severe SUI from mild/moderate SUI, binary logistic regression models were constructed, with the dichotomized Sandvik Severity Index as the dependent variable. This dichotomization was performed a priori to enable diagnostic accuracy analyses and the derivation of ROC-based cut-off values, which strictly require a binary reference standard. Age, body mass index and miscarriage were evaluated as potential covariates and included in the final models if they met the inclusion criterion (*p* < 0.20). Miscarriage was retained in the model because it showed a statistically significant association with SUI severity in the univariable analysis (*p* = 0.041) and has been previously linked to alterations in pelvic floor connective tissue and support in the literature [29].

Sensitivity, specificity, positive and negative likelihood ratios, and Youden’s index were calculated for each ultrasound parameter. The optimal cut-off value for the RVA at rest was determined using the Youden index and subsequently converted into degrees by solving the logistic regression equation, adjusted for age, BMI and miscarriage. Receiver operating characteristic (ROC) curves were generated, and the area under the curve (AUC) with 95% confidence intervals was calculated. Model calibration was assessed using the Hosmer–Lemeshow goodness-of-fit test and the Brier score.

Studentized residuals, leverage values, and Cook’s distances were examined to identify potential outliers and influential observations. There were no missing data for the index test or the reference standard. All statistical analyses were performed using SPSS Statistics version 29.0 (IBM Corp., Armonk, NY, USA). A two-tailed *p*-value < 0.05 was considered statistically significant.

### 2.5. Ethical Considerations

The study was conducted in accordance with the principles outlined in the Declaration of Helsinki (2013 revision) and the European Union General Data Protection Regulation 2016/679. The study protocol received ethical approval from the Research Ethics Committee of CEU Cardenal Herrera University (reference: CEEI25_646).

Written informed consent was obtained from all participants after they had received detailed information about the study aims, procedures, potential risks and benefits, and their right to withdraw at any time without consequence to their routine care. Data were anonymized using a unique alphanumeric code for each participant. No identifiable personal information was recorded in the study database, and all data were stored on password-protected servers with restricted access limited to the principal investigators.

Given the non-invasive nature of transperineal ultrasound, the study presented minimal risk to participants. No adverse events associated with the examinations were observed.

## 3. Results

**Figure 2:**
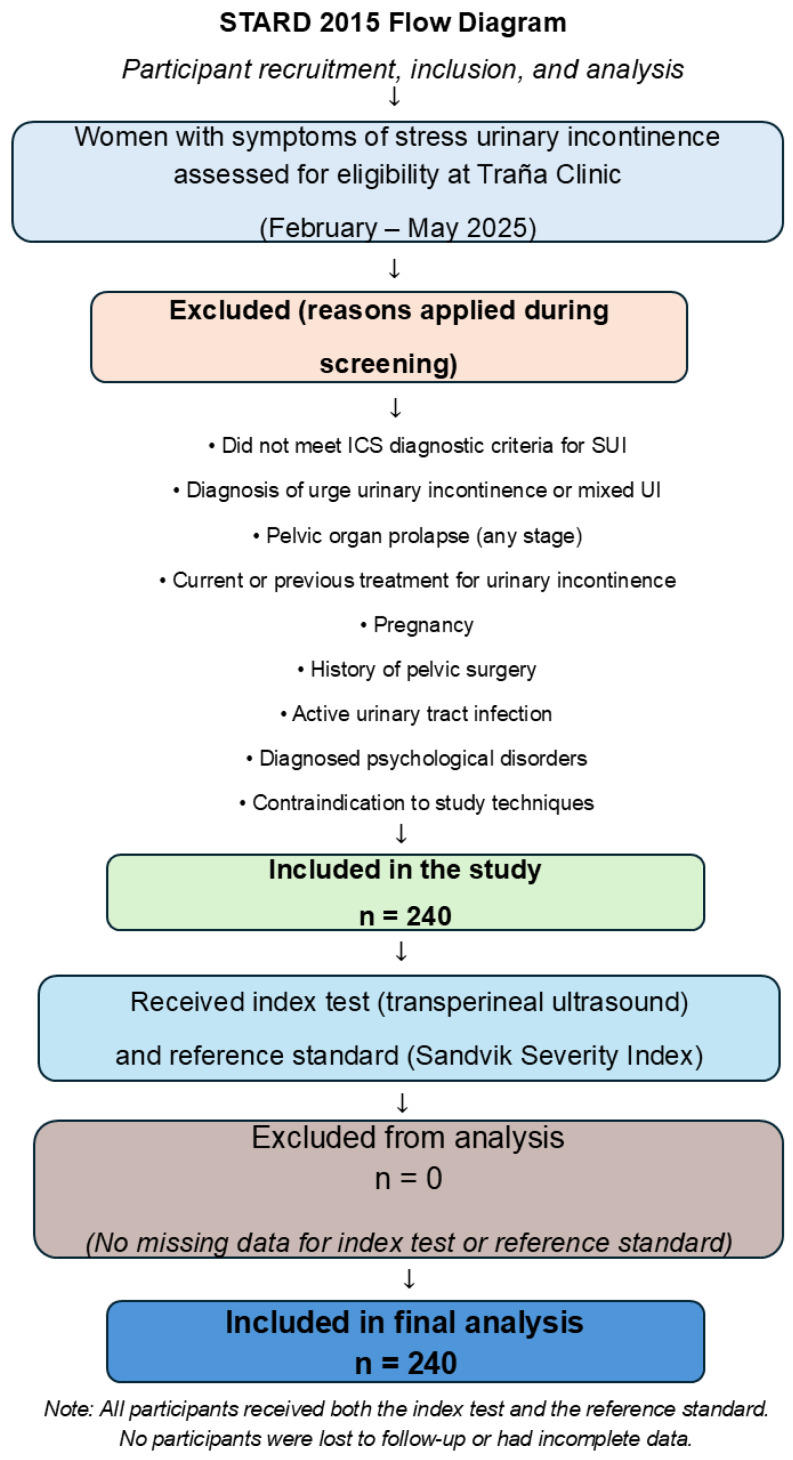
STARD 2015 flow diagram showing participant recruitment, inclusion, and analysis.

Figure 2 shows the flow of participants through the study according to the STARD 2015 guidelines. A total of 240 women with symptomatic stress urinary incontinence were included in the final analysis after applying the exclusion criteria. All participants underwent both the index test (transperineal ultrasound) and the reference standard (Sandvik Severity Index).

### 3.1. Participant Characteristics

The study included 240 women with SUI, with an average age of 41 years and slightly overweight (Table 1). The vast majority were non-smokers and did not use drugs, although 17.1% reported consuming alcohol. Classification by SUI severity on the Sandvik scale revealed comparable proportions of participants in the mild, moderate, and severe categories, with a markedly smaller proportion in the very severe category. In terms of obstetric characteristics, participants reported an average of 1.4 pregnancies, the majority being natural births (54.2%) and only four being multiple births. A caesarean section had been performed on 33.9%, 22.2% required an episiotomy, 17.5% reported perineal laceration, and 11.3% reported having had a miscarriage. When differentiating by degree of severity of SUI, no significant differences were observed in most of the variables related to childbirth, except for miscarriage, which occurred at a higher rate in the groups with milder SUI. Correlation analysis showed that severity assessed by Sandvik was not associated with age (*p* = 0.306), BMI (*p* = 0.119), or number of full-term pregnancies (*p* = 0.178). Although mean age differed significantly across the four SUI severity categories, Spearman correlation analysis revealed no significant monotonic association between age and the Sandvik severity score. This apparent difference arises because the group-level variations in age did not follow a consistent increasing or decreasing trend with higher severity levels.

### 3.2. Transperineal Ultrasound Findings and Relationship with SUI Severity

Ultrasound analysis showed a RVA at rest of 117.3° in the total population, which increased to 143.1° during exertion (Table 2). As the Sandvik severity grade increases, a greater opening angle is observed, both at rest (*p* < 0.001) and during exertion (*p* < 0.001). The correlation analysis showed a positive relationship between the severity calculated by the Sandvik scale and the value of the RVA evaluated at rest (ρ = 0.485, *p* < 0.001) or during exertion (ρ = 0.326, *p* < 0.001). The mobility assessment of the bladder neck showed that the BND in the total population was 22.6 mm, which is associated with significative hypermobility (>20 mm). There was a significant increase in BND values as the severity of SUI determined by Sandvik increased (*p* < 0.001). Correlation analysis confirmed this positive association between BND and the severity of SUI determined by Sandvik. The URA showed normal values (<20°) and was similar across the different severity grades as determined by the Sandvik classification.

**Figure 3:**
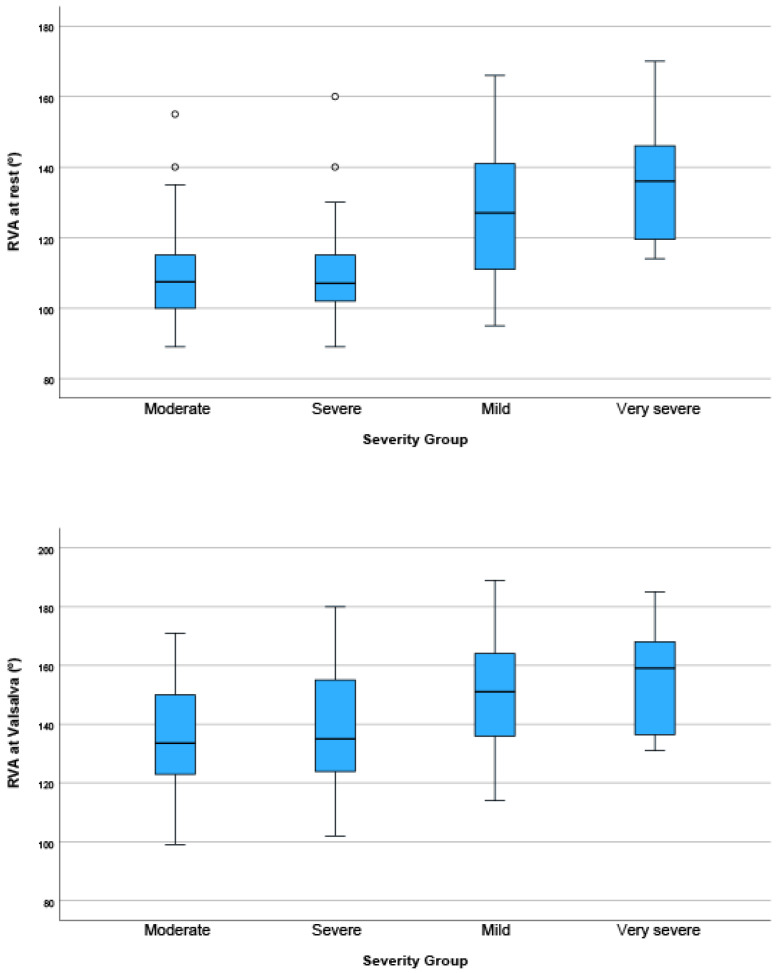
Boxplots showing the distribution of retrovesical angle (RVA) at rest (top panel) and during Valsalva manoeuvre (bottom panel) according to Sandvik Severity Index groups. The boxes represent the interquartile range (25th–75th percentiles), the horizontal line inside each box indicates the median, whiskers extend to 1.5 times the interquartile range, and individual data points are shown as outliers. Note the progressive increase in RVA values with higher severity grades, with some overlap between adjacent categories, particularly in milder groups.

The distributions of RVA values at rest and during Valsalva manoeuvre across Sandvik severity groups are illustrated in Figure 3, demonstrating a clear trend toward larger angles in more severe cases while highlighting inter-individual variability and overlap between categories.

Table 3 shows the discriminatory performance of the ultrasound measurements for distinguishing severe or very severe SUI. RVA at rest demonstrated the highest discriminatory capacity, with an AUC of 0.83 (95% CI: 0.77–0.89), high specificity (84%; 95% CI: 0.78–0.90), and acceptable sensitivity (71%; 95% CI: 0.60–0.80). This parameter also showed the closest performance to the maximum model that included all ultrasound variables.

To facilitate practical application in patients with average characteristics (41 years, BMI 25.6 kg/m^2^, 0.11 miscarriages), the following threshold for RVA at rest is established: a RVA of 118.1°, derived from the formula

$$\begin{array}{c} {RVA\;at\;rest}_{severity\;cut}=\frac{\ln\left(\frac{P}{1-P}\right)-\left(\beta_{0}+\beta_{1}\times Age+\beta_{2}\times BMI+\beta_{3}\times Miscarriage\right)}{\beta_{4}}\\ =\frac{\ln\left(\frac{0.373}{1-0.373}\right)-\left(-11.814+0.003\times 41.0+0.061\times 25.64-0.717\times 0.11\right)}{0.082}=118.1 \end{array}$$

where *P* represents the probability of the optimal cut-off point, β_0_ is the model constant, and β_1_, β_2_, β_3_, β_4_ are the coefficients for age, BMI, miscarriage and bladder neck opening, respectively. This approach preserves the adjustment for covariates while providing a clinically interpretable threshold.

### 3.3. Impact of SUI on HRQOL

The analysis of the impact of UI on HRQOL is shown in Table 4. The average impact assessed using the ICIQ-SF questionnaire was 12.0 ± 4.6 and increased significantly with the degree of severity determined using the Sandvik scale (*p* < 0.001). The correlation analysis showed that the severity assessed by Sandvik correlates positively with ICIQ-SF (ρ = 0.662, *p* < 0.001). None of the dimensions assessed by King’s Health Questionnaire correlated with the severity determined by Sandvik. The Symptom Severity Scale (SSS) increased with greater severity as determined by Sandvik (*p* = 0.003), as also confirmed by the correlation analysis (ρ = 0.243, *p* < 0.001).

The opening of the bladder neck evaluated by RVA correlated positively with the negative impact of SUI on HRQOL as determined by the ICIQ-SF, both at rest (ρ = 0.412, *p* < 0.001) and during exertion (ρ = 0.258, *p* = 0.012). However, only the role limitations correlated with RVA at rest (ρ = 0.162, *p* < 0.001 during exertion assessed using the KHQ (ρ = 0.147, *p* = 0.035), showing no relationship with any of the other dimensions assessed using the KHQ.

## 4. Discussion

This study is the first to quantify bladder neck opening using transperineal ultrasound both at rest and during the Valsalva manoeuvre. It demonstrates a clear, graded relationship between these measurements and SUI severity as classified by the Sandvik Severity Index. Furthermore, the ultrasound parameters showed significant associations with the impact of SUI on HRQOL.

The assessment of SUI severity using transperineal ultrasound has proven useful, although its discriminatory capacity is limited. The principal finding was that the RVA, also termed β-angle, measured at rest provided the strongest discriminatory capacity for distinguishing severe/very severe SUI from mild/moderate cases, achieving an AUC of 0.83, high specificity (84%) that minimises false positives, and acceptable sensitivity (71%). To our knowledge, there are no studies that measure the diagnostic capacity of ultrasound in classifying the severity of SUI in women, only the presence or absence of SUI, which limits direct comparison with our findings.

These results expand the existing literature, which has largely focused on the binary diagnosis of SUI rather than on graded severity. Keshavarz et al. [30] reported that a β-angle > 127° during Valsalva manoeuvre yielded an AUC of 0.89 for detecting SUI compared with continent women. Similarly, Turkoglu et al. [4] found high diagnostic performance for a change in α-angle > 16° and BND > 11.2 mm. Dong et al. [14] observed greater dynamic changes in RVA among women with SUI but no significant difference in URA, which is consistent with our findings.

Xiao et al. [16], using 3D translabial ultrasound, reported an AUC of 0.82 for dynamic parameters. However, their higher specificity (>90%) and lower sensitivity (<68%) differed from the more balanced performance observed in our study with RVA at rest. Similarly, Sendag et al. [24] reported that a posterior RVA > 120° is associated with poor bladder neck support, with sensitivities of 96% and 53% and specificities of 85% and 100%, respectively. Recent studies incorporating dynamic parameters (e.g., velocity of angle change during Valsalva) or artificial intelligence models have achieved AUC values of up to 0.87 [31,32,33]. For instance, Wang et al. [33] reported an AUC of 0.87 by combining the average speed of the β-angle, maximum speed of the URA, and Valsalva duration. Similarly, Chen et al. [34] obtained an AUC of 0.869 using an optimized DenseNet-121 deep learning model based on transperineal ultrasound images. However, most of these approaches continue to address binary classification (presence versus absence of SUI) rather than graded severity. The present work therefore fills a clinically important gap by demonstrating that a single, easily obtained resting measurement (RVA at rest) outperforms combined dynamic parameters and offers a practical threshold for identifying women who may require more aggressive management. Our study has established a cut-off point of 118.1° for bladder neck opening to identify severe/very severe cases of SUI, providing a novel contribution to the literature. Although the proposed formula can be applied to different ages, BMIs and miscarriages, its use is recommended within the age ranges of 26–68 and BMI 20–35 kg/m^2^ in this study, both defined by 5–95% percentiles to minimise the impact of extreme values. For populations outside these ranges, additional prospective validation is suggested.

Our results align with established ICIQ-SF severity categories [35,36]. The strong correlation between Sandvik severity and ICIQ-SF scores, together with the positive association between RVA and perceived HRQOL impact, reinforces the clinical value of RVA measurement. Recent evidence shows that conservative interventions targeting pelvic floor dysfunction and urogenital symptoms can lead to significant improvements in both symptom burden and quality of life [37]. While the KHQ provides useful domain-specific information [21], it lacks a formal severity classification for SUI and appears less sensitive than the ICIQ-SF for detecting graded differences in symptom severity and their anatomical correlates [38]. Consequently, the ICIQ-SF appears to be the more appropriate instrument when combining transperineal ultrasound assessment with patient-reported outcomes.

It remains uncertain whether similar associations between RVA at rest and SUI severity would be observed if objective measures such as pad testing or urodynamic studies had been used as the reference standard. Pad testing provides a quantitative measure of urine leakage volume, which may not correlate as strongly with anatomical parameters such as RVA. Urodynamic evaluation, on the other hand, could offer mechanistic insight by distinguishing between urethral hypermobility and intrinsic sphincter deficiency; the RVA might be expected to show a stronger relationship with hypermobility-related incontinence than with sphincter deficiency. Future studies incorporating these objective reference standards are warranted to better define the role of RVA measurement in severity assessment.

### 4.1. Strengths and Limitations

Strengths of this study include the relatively large sample size (n = 240) of women with symptomatic SUI, consecutive recruitment, and strict blinding between the index test (transperineal ultrasound) and the reference standard (Sandvik Severity Index). The study adhered to the STARD 2015 guidelines for reporting diagnostic accuracy studies, and all ultrasound measurements were performed using a standardized protocol with clear definitions of the RVA, BND, and URA. The identification of a clinically applicable cut-off value (118.1°) for the RVA at rest represents a practical contribution for severity stratification using a non-invasive, real-time, and widely available imaging technique.

Limitations should also be acknowledged. First, the proportion of participants with very severe SUI was modest (9.6%), which may have reduced statistical power for subgroup analyses in the most severe category. Second, although the RVA at rest demonstrated good discriminatory capacity (AUC 0.83, specificity 84%), the moderate sensitivity and specificity observed may partly reflect the multifactorial pathophysiology of SUI, including intrinsic sphincter deficiency not fully captured by morphological ultrasound parameters alone. Technical factors, such as variability in Valsalva manoeuvre effort and transducer positioning, may also have influenced results, as previously noted in the literature. Examinations were performed predominantly in the lithotomy position, and pelvic floor muscle function was not assessed. Finally, the proposed cut-off value was derived from a population with a mean age of 41 years and mean BMI of 26.5 kg/m^2^; therefore, external validation in broader age groups, BMI ranges, and diverse clinical settings is warranted.

Although the final sample in the very severe SUI category (n = 23) was smaller than originally planned, post hoc power analyses confirmed that the large observed effect sizes yielded power above 0.99 for all key comparisons. Sensitivity analyses merging the severe and very severe categories (n = 89) maintained statistically significant differences for RVA at rest, RVA at Valsalva, and BND (all *p* < 0.001), supporting the robustness of the graded associations reported.

A further methodological consideration concerns the dichotomization of the Sandvik Severity Index for the primary diagnostic accuracy analysis. Although dichotomization may reduce information from the original ordinal scale, analyses using the four original severity categories were also performed through Kruskal–Wallis tests and Spearman correlations to preserve the graded nature of the data.

Another important limitation of this study concerns the choice of the Sandvik Severity Index as the reference standard for grading SUI severity. Although this validated, symptom-based instrument demonstrates good criterion validity against objective pad-weighing tests [19] and showed strong correlation with the impact on HRQOL as measured by the ICIQ-SF in our cohort, it remains a brief patient-reported questionnaire and is therefore subject to potential reporting or recall bias. This introduces a risk of misclassification bias in the reference standard, which may have influenced the discriminatory performance of the ultrasound parameters for severity grading.

Furthermore, as a brief patient-reported tool, the Sandvik Index does not provide direct objective quantification of urine leakage volume nor detailed information on the underlying mechanisms of SUI (urethral hypermobility versus intrinsic sphincter deficiency). While urodynamic studies remain the reference standard for differentiating these mechanisms, they are invasive and not routinely indicated in straightforward cases managed conservatively. Pad testing, although objective, is time-consuming and may not be practical in all clinical contexts. Blinding between the index test and the reference standard was maintained to minimise differential misclassification. Validated symptom severity indices such as the Sandvik Index are nevertheless commonly employed in pragmatic diagnostic accuracy studies when the aim is to stratify clinically meaningful severity grades. Future studies could further strengthen the reference standard by incorporating objective measures (e.g., standardised pad testing) or latent class analysis when multiple imperfect references are available.

Several potentially relevant confounding factors were not assessed in this study. Menopausal status and hormonal therapy (systemic or vaginal) can significantly influence tissue quality, collagen turnover, and urethral support, thereby affecting both retrovesical angle measurements and SUI severity. Although age was included as a covariate in the adjusted models, explicit menopausal status was not recorded. Similarly, a detailed history of previous pelvic floor muscle training (beyond exclusion of recent treatment) was not collected; such training may improve muscle function and partially mitigate the impact of anatomical changes. Chronic respiratory disease or persistent cough, which causes repeated increases in intra-abdominal pressure, is a known contributor to worsening urethral hypermobility and SUI severity but was not evaluated. These unmeasured factors may have introduced residual confounding. Nevertheless, the primary associations between ultrasound parameters and SUI severity remained statistically significant after adjustment for the available covariates. Future diagnostic accuracy studies should incorporate systematic collection of menopausal status, hormonal therapy use, pelvic floor muscle training history, and respiratory conditions to allow more complete multivariable analysis.

A further limitation concerns the generalizability of the ultrasound findings. All transperineal ultrasound examinations and offline measurements were performed by a single highly experienced operator (>5 years of dedicated experience in pelvic floor ultrasound) in a specialized pelvic floor physiotherapy clinic. Although previous validation studies have demonstrated excellent intra- and inter-observer reliability for the measurement of RVA, BND, and URA when standardized protocols are followed, the diagnostic performance observed in this study may not be directly reproducible by less experienced operators or in routine clinical practice without adequate training. The learning curve associated with transperineal ultrasound, particularly for accurate identification of the bladder neck and measurement of the RVA, should not be underestimated. Therefore, while the technique is non-invasive and uses widely available equipment, its implementation in general gynecological or urological settings would require structured training and quality assurance processes to achieve comparable diagnostic accuracy. Future multicenter studies involving operators with varying levels of experience are needed to confirm the external validity and reproducibility of the RVA at rest as a severity marker in broader clinical contexts.

Finally, the proposed 118.1° RVA cut-off was derived and evaluated within the same cohort. Lacking internal or external validation, its diagnostic accuracy may be subject to optimism bias. Thus, this threshold is a preliminary estimate requiring prospective external validation before clinical implementation.

### 4.2. Clinical Implications

Transperineal ultrasound measurement of the RVA at rest may serve as a useful objective adjunct to clinical history, physical examination, and validated symptom questionnaires in the assessment of women with SUI. With high specificity (84%) for detecting severe or very severe SUI, an RVA at rest ≥ 118.1° could help identify patients with more significant anatomical urethral support defects, potentially supporting earlier referral to specialized pelvic floor clinics or more intensive conservative management strategies in selected cases. Conversely, a normal RVA in patients reporting severe symptoms might prompt clinicians to consider other contributing factors, such as intrinsic sphincter deficiency, and adjust counseling accordingly. Providing patients with an objective ultrasound measurement of bladder neck opening may also facilitate shared decision-making by offering a clearer anatomical explanation of their symptoms and their potential impact on quality of life. However, the proposed cut-off value of 118.1° should be regarded as exploratory and hypothesis-generating, as it was both derived and evaluated within the same study cohort. External validation in independent populations is therefore required before this parameter can be considered for influencing clinical decision-making, including referral patterns or the choice between conservative and surgical treatment. Prospective studies are needed to determine whether incorporation of RVA measurement into clinical pathways can meaningfully improve patient selection, counseling, or outcomes.

## 5. Conclusions

Transperineal ultrasound measurement of the RVA at rest provides a reliable, non-invasive method for grading SUI severity, with good discriminatory capacity and correlation with HRQOL impact. These findings support its potential as an adjunct to clinical assessment, although prospective validation in independent cohorts is required to confirm its utility in guiding management decisions.

These findings indicate that transperineal ultrasound measurement of the RVA at rest has good discriminatory capacity (AUC 0.83, specificity 84%) for identifying women with severe or very severe SUI. The parameter may have potential as an adjunct to clinical assessment for severity stratification and could help identify women who might warrant closer evaluation or specialist referral. However, given the moderate sensitivity (71%) and the absence of data linking its use to improved management outcomes, prospective studies evaluating clinical utility and externally validating the proposed cut-off in independent cohorts are required before it can be recommended for routine screening or referral decisions.

Future multicentre studies with broader populations are needed to validate the proposed cut-off value across different age and BMI ranges and to explore the added value of combining transperineal ultrasound with functional or artificial intelligence-based approaches.

## Figures and Tables

**Table 1:** Anthropometric, obstetric, and lifestyle characteristics of participants according to stress urinary incontinence severity assessed by the Sandvik Index.

	SUI					*p*
	Total	Mild	Moderate	Severe	Very Severe	
n (%)	240 (100)	66 (27.5)	85 (35.4)	66 (27.5)	23 (9.6)	
Age	41.0 ± 12.2	42.3 ± 13.5	38.2 ± 9.7	43.5 ± 12.9	40.2 ± 12.2	0.042
BMI	26.6 ± 4.7	25.8 ± 4.8	24.6 ± 3.9	25.9 ± 4.7	28.2 ± 6.2	0.065
Smoker						
No	235 (97.9)	63 (95.5)	84 (98.8)	66 (100)	22 (95.7)	0.232
Yes	5 (2.1)	3 (4.5)	1 (1.2)	0 (0)	1 (4.3)	
Alcohol						
No	199 (82.9)	56 (84.8)	67 (78.8)	57 (86.4)	19 (82.6)	0.082
Yes	41 (17.1)	10 (15.2)	9 (13.6)	9 (13.6)	4 (17.4)	
Drugs						
No	236 (98.3)	63 (95.5)	84 (98.8)	66 (100)	23 (100)	0.174
Yes	4 (1.7)	3 (4.5)	1 (1.2)	0 (0)	0 (0)	
Full-term pregnancies	1.4 ± 1.1	1.3 ± 1.2	1.4 ± 1.1	1.4 ± 1.1	1.7 ± 1.0	0.398
Natural birth						
No	130 (54.2)	41 (62.1)	44 (51.8)	38 (57.6)	7 (30.4)	0.060
Yes	110 (45.8)	25 (37.9)	41 (48.2)	28 (42.4)	16 (69.6)	
Multiple birth						
No	235 (98.3)	66 (100)	83 (97.6)	64 (98.5)	22 (95.7)	0.500
Yes	4 (1.7)	0 (0)	2 (2.4)	1 (1.5)	1 (4.3)	
Caesarean section						
No	158 (66.1)	44 (66.7)	56 (65.9)	43 (66.2)	15 (65.2)	0.999
Yes	81 (33.9)	22 (33.3)	29 (34.1)	22 (33.8)	8 (34.8)	
Episiotomy						
No	186 (77.8)	49 (74.2)	65 (83.1)	54 (83.1)	18 (78.3)	0.654
Yes	53 (22.2)	17 (25.8)	20 (23.5)	11 (16.9)	5 (21.7)	
Perineal laceration						
No	198 (82.5)	53 (80.3)	73 (85.9)	53 (80.3)	19 (82.6)	0.773
Yes	42 (17.5)	13 (19.7)	12 (14.1)	13 (19.7)	4 (17.4)	
Miscarriage						
No	213 (88.8)	58 (87.9)	70 (82.4)	62 (93.9)	23 (100)	0.041
Yes	27 (11.3)	8 (12.1)	15 (17.6)	4 (6.1)	0 (0)	

Data shown as mean ± standard deviation or number of responses (percentage). *p* value calculated by Chi square test for categorical variables and the Kruskal–Wallis test for quantitative variables. BMI, body mass index.

**Table 2:** Transperineal ultrasound parameters according to stress urinary incontinence severity assessed by the Sandvik Index.

	Sandvik					*p*
	Total	Mild	Moderate	Severe	Very Severe	
RVA at rest (°)	117.3 ± 17.7	110.1 ± 13.1	109.5 ± 11.2	127.6 ± 18.6	136.6 ± 17.1	<0.001
RVA at Valsalva (°)	143.1 ± 19.5	136.6 ± 17.8	138.7 ± 18.9	150.9 ± 18.0	155.4 ± 17.9	<0.001
ΔRVA (°)	25.8 ± 15.5	26.6 ± 13.0	29.2 ± 15.7	23.1 ± 14.8	18.6 ± 19.7	0.035
BND (mm)	22.6 ± 10.8	21.8 ± 9.8	19.4 ± 8.7	25.2 ± 12.6	28.9 ± 11.1	<0.001
URA (°)	15.6 ± 8.5	15.2 ± 5.4	15.1 ± 6.1	16.7 ± 11.6	15.1 ± 12.7	0.851

Data shown as mean ± standard deviation. *p* value calculated by Kruskal–Wallis test. RVA: retrovesical angle. ΔRVA = RVA at Valsalva manoeuvre—RVA at rest; BND, Bladder Neck Descent, BND = BSD at rest—BSD at Valsalva (BSD, Bladder-Symphysis Distance); URA, Urethral Rotation Angle.

**Table 3:** Discriminatory performance of transperineal ultrasound parameters for distinguishing severe or very severe stress urinary incontinence (Sandvik Index as reference standard).

Variables ^a^	AUC [CI 95%]	Se [CI 95%]	Sp [CI 95%]	LRp [CI 95%]	LRn* [CI 95%]	P Fit HL	BS
RVA at rest (°)	0.83 [0.77, 0.89]	0.71 [0.60, 0.80]	0.84 [0.77, 0.90]	4.45 [3.01, 6.58]	0.35 [0.25, 0.48]	0.691	0.154
RVA at Valsalva (°)	0.74 [0.67, 0.80]	0.85 [0.76, 0.92]	0.54 [0.46, 0.62]	1.87 [1.54, 2.27]	0.27 [0.16, 0.45]	0.361	0.198
ΔRVA (°)	0.69 [0.62, 0.76]	0.48 [0.38, 0.59]	0.81 [0.74, 0.87]	2.61 [1.75, 3.88]	0.63 [0.51, 0.79]	0.336	0.209
BND (mm)	0.64 [0.57, 0.72]	0.69 [0.58, 0.78]	0.55 [0.47, 0.63]	1.52 [1.21, 1.91]	0.57 [0.41, 0.80]	0.120	0.219
URA (°)	0.69 [0.62, 0.76]	0.57 [0.46, 0.68]	0.72 [0.64, 0.79]	2.06 [1.51, 2.82]	0.59 [0.46, 0.77]	0.943	0.207
Maximum model	0.84 [0.79, 0.89]	0.75 [0.65, 0.84]	0.83 [0.76, 0.88]	4.37 [3.02, 6.33]	0.30 [0.21, 0.43]	0.922	0.149

RVA: retrovesical angle. ΔRVA = RVA at Valsalva maneuver—RVA at rest; BND, Bladder Neck Descent, BND = BSD at rest—BSD at Valsalva (BSD, Bladder-Symphysis Distance); URA, Urethral Rotation Angle; AUC = area under the ROC curve; Se = sensibility; CI = confidence interval; Sp = specificity; LRp = positive likelihood ratio; LRn* = the inverse of negative likelihood ratio to allow for a direct comparison with LRp; P fit HL = Hosmer–Lemeshow goodness-of-fit test; BS = Brier Score, metric of prediction accuracy; BMI = body mass index. *p* value > 0.05 indicates a good fit. ^a^ Corrected by age, BMI and miscarriage.

**Table 4:** Impact of stress urinary incontinence on HRQOL according to severity assessed by the Sandvik Index.

	SUI					*p*
	Total	Mild	Moderate	Severe	Very Severe	
ICIQ-SF	12.0 ± 4.6	8.8 ± 3.6	10.1 ± 3.7	16.1 ± 2.8	16.4 ± 2.0	<0.001
King’s Health Questionnaire						
General health perceptions	73.7 ± 20.1	70.8 ± 22.2	73.2 ± 19.6	77.3 ± 16.8	72.8 ± 23.7	0.406
Impact on life	48.1 ± 32.5	49.5 ± 34.7	46.3 ± 33.8	47.5 ± 29.3	52.2 ± 31.5	0.833
Role limitations	14.2 ± 22.3	11.9 ± 19.6	10.0 ± 15.0	17.2 ± 26.8	28.3 ± 31.2	0.048
Physical limitations	28.0 ± 28.2	24.2 ± 24.3	25.9 ± 28.7	29.8 ± 27.8	41.3 ± 34.8	0.151
Social limitations	10.0 ± 20.5	8.3 ± 18.2	8.1 ± 17.8	11.3 ± 19.6	18.4 ± 33.9	0.294
Personal relationships	14.0 ± 25.6	14.1 ± 26.5	11.6 ± 22.6	16.4 ± 27.7	15.2 ± 27.9	0.689
Emotions	22.5 ± 28.9	20.7 ± 27.4	19.6 ± 28.7	28.1 ± 30.5	21.7 ± 28.5	0.141
Sleep/Energy	20.3 ± 29.6	17.4 ± 26.7	20.4 ± 30.6	21.7 ± 30.5	23.9 ± 32.1	0.696
SSS	6.1 ± 4.9	4.9 ± 4.4	5.6 ± 4.5	7.4 ± 5.4	7.6 ± 4.8	0.003

Data shown as mean ± standard deviation. *p* value calculated by Kruskal–Wallis test. SSS, Symptom Severity Scale.

## Data Availability

To enhance transparency and ensure compliance with best scientific practices, we would like to clarify that the dataset supporting the findings of this study is publicly available in Zenodo. The dataset can be accessed without restrictions via the following DOI: https://doi.org/10.5281/zenodo.21371418.

## Abbreviations

The following abbreviations are used in this manuscript:

AUC	Area Under the Curve
BMI	Body Mass Index
BND	Bladder Neck Descent
BSD	Bladder–symphysis distance
CI	Confidence Interval
HRQOL	Health-Related Quality of Life
ICIQ-SF	International Consultation on Incontinence Questionnaire-Short Form
ICS	International Continence Society
KHQ	King’s Health Questionnaire
P	*p*-value
ρ	Coefficient of determination
ROC	Receiver operating characteristic
RVA	Retrovesical Angle
SSS	Symptom Severity Scale
SUI	Stress Urinary Incontinence
UI	Urinary Incontinence
URA	Urethral Rotation Angle alpha
